# Identification of Bicycling Periods Using the MicroPEM Personal Exposure Monitor

**DOI:** 10.3390/s19214613

**Published:** 2019-10-23

**Authors:** Robert Chew, Jonathan Thornburg, Darby Jack, Cara Smith, Qiang Yang, Steven Chillrud

**Affiliations:** 1 RTI International, Research Triangle Park, NC 27709, USA; jwt@rti.org; 2 Mailman School of Public Health, Columbia University, New York, NY 10032, USA; dj2183@cumc.columbia.edu (D.J.); cms2308@cumc.columbia.edu (C.S.); 3 Lamont-Doherty Earth Observatory, Columbia University, Palisades, NY 10964, USA; qyang@ldeo.columbia.edu (Q.Y.); chilli@ldeo.columbia.edu (S.C.)

**Keywords:** human activity recognition, machine learning, wearable sensors, exposure assessment, air pollution

## Abstract

Exposure assessment studies are the primary means for understanding links between exposure to chemical and physical agents and adverse health effects. Recently, researchers have proposed using wearable monitors during exposure assessment studies to obtain higher fidelity readings of exposures actually experienced by subjects. However, limited research has been conducted to link a wearer’s actions to periods of exposure, a necessary step for estimating inhaled dosage. To aid researchers in these settings, we developed a machine learning model for identifying periods of bicycling activity using passively collected data from the RTI MicroPEM wearable exposure monitor, a lightweight device capable of continuously sampling both air pollution levels and accelerometry parameters. Our best performing model identifies biking activity with a mean leave-one-session-out (LOSO) cross-validation F1 score of 0.832 (unweighted) and 0.979 (weighted). Accelerometer derived features contributed greatly to the model performance, as well as temporal smoothing of the predicted activities. Additionally, we found competitive activity recognition can occur with even relatively low sampling rates, suggesting suitability for exposure assessment studies where continuous data collection for long periods (without recharge) are needed to capture realistic daily routines and exposures.

## 1. Introduction

Efficiently mitigating human exposure to air pollution is a top public health objective. According to the most recent global burden of disease/comparative risk assessment, exposure to air pollution causes over 6.8 million premature deaths per year, with roughly equal contributions from ambient and household air pollution [1]. While many countries acknowledge this danger and have enacted air pollution prevention laws, developing specific rules and regulations to implement these laws can be challenging when the relationships between pollutant exposure and health effects are underspecified. Better information on exposure response relationships (how clean is clean enough?) can help regulators more effectively balance the often competing objectives of economic growth and public health.

In air pollution epidemiology, exposure assessment studies are the primary means for understanding links between exposure to chemical and physical agents and adverse health effects. However, exposure assessment studies have traditionally used imperfect proxies based on central site data to assess these relationships, even though ambient concentrations are poor predictors of personal exposure [2]. For measuring personal exposure, ambient concentration readings from stationary sensors have many drawbacks. First, people move through multiple indoor and outdoor micro-environments with fine-scale spatial and temporal heterogeneity in concentrations, resulting in substantial person-to-person variation even among individuals living within the same building or neighborhood. Stationary sensors are unable to capture these differences in daily routines and resulting exposures, potentially biasing health effect estimates. In addition, due to their fixed nature, stationary sensors are unable to decouple the spatiotemporal contributions of individual sources to a person’s exposure. Finally, the actual inhaled dose of a pollutant is a function of respiration rate, and, thus, of physical activity patterns. Since physical activity protects against many pollution-sensitive health outcomes, this may again confound average concentration-based estimates.

To help address concerns with air pollution exposure assessment studies based on fixed, centrally located sensor data, researchers have proposed using wearable monitors to more accurately capture personalized pollutant exposures. Unlike stationary sensors, personal monitors can better capture exposures actually experienced by the wearers and provides the spatial and temporal heterogeneity necessary to design interventions incorporating human behaviors and lifestyle [3]. Furthermore, if personal exposure data is combined with respiration data, it becomes possible to estimate potential inhaled dose, a much higher fidelity measure for mapping the exposure response than using device concentrations alone [4]. However, passively measuring respiratory rates is not always possible without subjects wearing additional devices, increasing the level of study complexity and increasing the risk of protocol non-compliance.

In this study, we focus on classification methods for determining levels of high activity directly from wearable exposure monitor data. This information can be used to develop tiers of respiratory rates for potential inhaled dose calculations, using the same wearable device required for personal exposure monitoring. Specifically, we develop a machine learning model for identifying periods of bicycling activity using passively collected data from the RTI MicroPEM wearable exposure monitor [5], a lightweight device capable of continuously sampling both particulate matter concentration and the wearer’s acceleration.

### 1.1. Motivation

Our focus on identifying periods of bicycling activity is motivated by the elevated risk of urban cyclists for pollutant exposure when compared to other urban residents. Since urban cyclists undertake strenuous exercise in close proximity to traffic, their potential dose is likely to be higher than many other urban residents, due both to their increased respiration rates and their proximity to traffic sources. For example, Zuurbier et al. [6] estimated cyclists inhale twice as much ultra fine particle (UFP counts) and PM2.5 (mass) per unit time as car and bus occupants because of their much higher minute ventilation, even though the measured UFP and PM concentrations were higher inside the vehicles. A similar study found the average daily dose of cyclists was 1.06–1.30 times higher than non-cyclists [7]. This larger inhaled dose is likely due to the average minute ventilation of cyclists being 4.3 times higher than vehicle passengers [8]. In terms of potential exposures, recent studies in major North American cities have found average exposures along bike routes to be higher than comparable fixed sidewalk stations [9] and significant associations between higher PM2.5 emissions and bicycling volume [10]. Pedestrians and joggers, in addition to cyclists, receive higher potential inhaled doses of traffic related pollutants, but cyclists are the preferred cohort because the exposure and health assessment equipment is less burdensome for cyclists to carry. Lastly, given the growth in ridership and infrastructural support for urban cycling in many U.S. cities [11], understanding cyclist exposure patterns can help address many practical issues of concern to the public. Does biking to and from work affect cardiovascular health or indicators of future health outcomes? Should one limit exercise and biking to the less polluted days or less polluted areas of the city? Do the health benefits of commuting by bicycle outweigh the costs?

### 1.2. Related Work

Human activity recognition (HAR), the task of identifying human actions from streaming data sources, has developed a broad literature encompassing various activities, devices, and data modalities (the most popular being video [12] and wearable sensors [13]). Given this diversity, we focus our review on only comparable studies that use accelerometer data from wearable sensors to identify periods of cycling activity. Parkka et al. [14] recorded data using a suite of different sensors, capturing readings such as skin temperature, chest accelerations, and audio in a naturalistic setting. They developed custom decision tree, automatic decision tree, and artificial neural network models to classify seven activity types, reporting their highest accuracy of 82% for identifying indoor cycling activity (on an exercise bike). In contrast, Bao and Intille [15] used only accelerometer data from five different areas of the body (right hip, dominant wrist, non-dominant upper arm, dominant ankle, and non-dominant thigh) in a semi-naturalistic setting. They reported an accuracy of 96.29% for bicycling recognition using a C4.5 tree based classifier over 20 activity classes, finding that thigh and wrist readings contributed the most to improved model performance. Riboni and Bettini [16] recorded accelerometer readings from Android HTC mobile devices (worn in the left pocket) and Sun SPOT sensors (wrist) to record 10 activity types in a semi-naturalistic setting. Using a hybrid statistical-ontological algorithm called COSAR-hist, the authors correctly identified periods of cycling with 92.04% precision and 99.52% recall. Yang [17] also used built-in mobile phone accelerometers to capture six activity types, finding accuracies ranging from 84.4% to 91.7% using a decision tree classifier on different subsets of vertical and horizontal accelerometer derived features. By adding a hidden Markov model to temporally smooth the decision tree’s predicted output, the overall accuracy increased by 6.25%.

While similar in many ways to existing approaches, our work contributes to the literature on several fronts. First, our results provide supporting evidence that HAR modeling based on mobile phone sensors and custom lab platforms extend to wearable devices for personal pollutant exposure. In addition to accelerometer data, the RTI MicroPEM also passively captures and records temperature, relatively humidity and particle concentration, helping us understand if these streams provide marginal benefit to activity recognition tasks. Second, we used a low sampling rate of one reading per 30 seconds to model bicycling activity. While it is uncommon in the HAR literature to use such a low sampling rate, such low rates are common when using wearable environmental monitoring devices to extend battery life, since continuous data collection for longer periods (24+ hours) are often needed to capture realistic representation of daily routines and exposures patterns. To our knowledge, this work is the first to test the feasibility of HAR models on conditions and devices germane to exposure assessment studies, providing necessary evidence and context for air pollution epidemiologists and environmental regulators.

## 2. Data

### 2.1. Device Description

The air pollution sensing device used in this study is the RTI MicroPEM v3.2b (RTI International, Research Triangle Park, NC, USA). The RTI MicroPEM is a personal aerosol exposure monitor that weighs 225 g, has a volume of 510 cm^3^, and has been successfully deployed in exposure assessment studies using lanyards, waist packs, shoulder harnesses, and camera cases. The MicroPEM uses a validated dual stage sharp cut point impactor inlet to collect either PM2.5 or PM10. Key capabilities of the system include: (a) providing both integrated PM samples on a 25 mm Teflon filter, and real-time PM data by nephelometry at 780 nm for periods as short as 1 s, (c) an on-board Oki ML 8953 (Oki Group, Tokyo, Japan) three-axis accelerometer with a three gravity unit (GU) range and nominal resolution of 0.02 GU, (c) collecting QC data such as pump flow, filter pressure drop, battery voltage, temperature, relative humidity, and wearing compliance level on-board to subsequently validate the PM data, and (d) allowing fully programmable operational control and timing of system on/off cycling, nephelometer data capture rate, and accelerometer output capture rate.

### 2.2. Data Collection

Participant data was collected by Columbia University as part of a pilot grant on potential inhaled dose, biking, and cardiovascular indicators. Participants were those who bicycle commute to work and were recruited via local media stories. Prospective participants submitted interest forms and were selected based on length of ride (45 minutes ± 15), non-smoking status, use of smartphone, and no history of heart disease.

Participants were expected to complete up to five non-consecutive 24-hour monitoring sessions within a two-week window. The 24-hour period began in the morning and ended the following morning. Sessions had to contain at least one bike commute either to or from work. Two RTI MicroPEMs were worn in the front pockets of a hydration vest. Vest with monitors were to be worn outside all clothing during the day and participants were instructed to hang the vest with the monitors on a doorknob in their room while they slept. Participants logged each of their bicycle rides with the equipment using Strava phone-based GPS tracking app.

### 2.3. Dataset Description

The data for this study were generated from seven participants for a total of 21 unique sessions ranging from roughly 24–27 h during the fall of 2016. To prevent overly optimistic model evaluation metrics, only one set of MicroPEM readings per session were used for the analysis. Unless there were known issues with one the MicroPEM readings, the devices used for modeling were randomly chosen among the two available per session. In all sessions, a minority of observations are biking, ranging from 3.1% to 11.8% per session. The implications of this class imbalance is discussed further in Section 3.2 and Section 4. Table 1 summarizes the number of records, outcome balance, and raw sensor measurements across the sessions. Note that the record counts and percent biking reflect the number of observations after the pre-processing steps described in following section.

### 2.4. Data Preprocessing and Feature Creation

The dataset contains observations sampled from the RTI MicroPEM every 30 seconds. While accelerometer data is recorded whenever the RTI MicroPEM takes a reading, the temperature, relative humidity, and nephelometer readings are only recorded once every minute. When these features were not recorded by the device, we used linear interpolation to estimate the intermediate values. In addition, all features were mean centered and scaled to the unit variance to help standardize the value ranges across variables.

To generate model features, we created a sliding window of six time steps with a corresponding step size of 3. The resulting sliding windows contain 50% overlap in observations with neighboring windows, a well-established convention in the HAR literature [18,19]. The main motivation for this approach is that the overlap prevents missing events that would occur at the boundaries between two neighboring windows if using non-overlapping windows. To help capture the distributional characteristics, we calculated a total of nine summary statistics (mean, median, max, min, standard deviation, skew, kurtosis, 20% percentile, and 80% percentile) for observations contained within each sliding window. This process was repeated for each of the six measurements recorded by the RTI MicroPEM (accelerometer (X-, Y-, Z-axes), temperature, relative humidity, RH-corrected nephelometer), resulting in a total of 54 features.

## 3. Methods

### 3.1. Biking Recognition Models

To develop HAR models for biking activity, we assessed several classification algorithms with different underlying modeling assumptions to get diverse biking predictions. Specifically, we developed an L1-regularized logistic regression [20], a K-nearest neighbors classifier [21], and a gradient boosted trees classifier [22] using the scikit-learn library in Python [23]. Logistic regression is a parametric statistical model that assumes that a categorical outcome can be described by linear combination of covariates (**x**) and associated weights (**w**). In the case of binary classification, the functional form for this relationship can be summarized as:

$$logit\left(p\right)=\ln\left(\frac{p_{i}}{1-\;p_{i}}\right)=w^{T}x_{i}$$

where $p_{i}$ is the probability that outcome is positive (in our case, “biking”), ***x_i_*** is a vector of covariate values for observation ***i*** and **w** is a vector of weights shared across all observations, learned from the data. Logistic regression is often a popular modeling choice due to it being fast to train, having weights that are straightforward to interpret when modeling assumptions are met, and it being easy to incorporate non-linear decision boundaries, multi-class classification, and regularization to penalize overly complex models.

K-nearest neighbors is a non-parametric algorithm that assumes new observations will have similar outcomes to past observations that share similar covariate values. To operationalize this idea, a new observation is compared with up to *K* neighboring observations, with the most common outcome amongst the K neighbors being assigned to the new observation. Neighbors are determined by calculating the pointwise distance between the new observation and other observations in the training data, where the researcher specifies the distance metric and the number of neighbors eligible for comparison (*K*). While the algorithm is intuitive to understand and explain, its accuracy as a classifier can be severely compromised by noisy data or irrelevant features.

The gradient boosted trees method uses gradient boosting to improve the quality of fit for an ensemble of weakly predictive decision trees. An individual decision tree is designed to partition the joint covariate space into disjoint regions of high outcome class purity (e.g., disjoint regions that each contain either mostly “biking” or “not biking” exclusively, instead of regions containing a balanced mix of “biking” and “not biking”). Since learning an optimal decision tree is NP-complete, algorithms for constructing classification trees are locally greedy when considering splits. This, combined with the tendency for overly complex decision trees to overfit, motivates the demand for ensemble methods combining predictions from several less complex classification trees. Boosting is an ensemble method that adds new classifiers iteratively based on which observations were most difficult to classify by previous classifiers in the ensemble. When adding a new classifier to the ensemble, the method gives more weight to observations that previous classifiers found difficult to categorize, focusing the new model on areas where previous models struggled. Gradient boosting reframes boosting as an optimization problem, where the objective to minimize a loss function. For gradient boosted trees, this results in adding new trees that help minimize the loss in an iterative fashion; the construction of the new tree is based on feedback from the functional gradient descent mechanism that drives the optimization routine.

To synthesize the perspectives each model brings to the classification task, we also developed a stacked ensemble model using the predictions from the three previously mentioned algorithms. Stacked ensembling, alternatively called “Super Learning” in the statistical learning literature [24], is a method of combining predictions of several diverse models to create a single composite prediction. Traditional ensembling methods for classification usually takes the majority predicted class across all component classifiers to determine new predictions. In contrast, stacked ensembles use the model predictions as input features for a new classifier, creating a weighted contribution of each base model. In addition to often exhibiting good performance empirically, stacked ensembles have been proven to perform asymptotically as well as or better than the best base model used in the ensemble [25]. Our stacked ensemble takes the predicted probabilities of the three base models (logistic regression, K-nearest neighbors, and the gradient boosted tree) and uses them as input variables for a new gradient boosted tree.

As a final approach, we temporally smoothed the predictions from the stacked ensemble to reduce noise among neighboring observations. Conceptually similar to smoothing and filtering operations in statistics and signal processing, this operation takes a sliding window of adjacent predictions and returns the majority predicted class within that set of observations. The intuition for why we explored this approach is that it helps prevent spastic changes in predictions over short periods of time, since (1) subjects tend not to switch activity categories at a high frequency under normal circumstances and (2) short periods of activity are less likely to change a person’s inhalation rate, the ultimate next step in this line of research. We refer to this simple approach as the “mode smoothed transformer” in subsequent sections. For our implementation, we used a sliding window of 11 observations, centered on the current observation (the current observation, plus five observations before and after). To determine settings that perform well on the training set, we performed hyperparameter tuning for each classifier, resulting in a single model representation for each algorithm (final model hyperparameters are documented in Table A1 of the Appendix A).

### 3.2. Model Evaluation

To help assess the validity of the findings, we ran a leave-one-session-out (LOSO) cross-validation strategy, training the models on all but one session and assessing model performance on the remaining hold-out session. This process is repeated so that each session is used as a hold-out, resulting in *S* runs per model, where *S* is the number of sessions in the dataset. This strategy prevents observations from the same session occurring in both the training and test sets, a well-documented scenario that often leads to overfitting and poor generalization on new data [18,19,26]. An additional benefit of this validation approach is that it most closely mimics the process of adding new sessions to exposure assessment studies, since we assume that new sessions will not have activity labels for their passively recorded sensor data.

For each validation fold, we calculate the precision, recall, and F1-score, both unweighted and weighted by the number of true instances for each label to help account for the label imbalance (i.e., the greater occurrence of “non-biking” to “biking” observations in our dataset) [23]. Lastly, the mean and standard deviation across all hold-out session folds are reported for each combination of classification model and metric to provide an indication of variability across sessions.

## 4. Results

### 4.1. Model Performance

Table 2 summarizes the LOSO cross-validation metrics across the various biking recognition models. The best performing model was the mode smoothed transformer, showing a mean LOSO cross-validation F1 score of 0.832 (unweighted) and 0.979 (weighted). When examining the components of the F1, the mode smoothed transformer also tended to perform better in both precision and recall; only the logistic regression model had a better mean recall (unweighted), though it also suffered from the lowest mean precision across all models.

Note that the standard deviation of the unweighted metrics is fairly large across folds, ranging from 6.3% to 19.3%. We believe this variation may in part be driven by the range in class imbalance between sessions, which the weighted metrics account for. To illustrate this effect, we calculated the Pearson correlation coefficient between (1) the percent of observations labeled “biking” per session and (2) each evaluation metrics per session. At the session-level, the unweighted metrics were considerably correlated with class imbalance (F1: 0.53; precision: 0.48; recall: 0.47), whereas the weighted metrics were nearly uncorrelated (F1: –0.18; precision: –0.19; recall: –0.23). We also ran paired sample t-tests of no correlation (null hypothesis: true correlation is equal to zero) to help take into account sample size on the relationship. The unweighted metrics were found to be significantly correlated with percent biking at an alpha of 0.05 (F1: p = 0.012; precision: p = 0.026; recall: p = 0.030), whereas the weighted metrics were not (F1: p = 0.435; precision: p = 0.411; recall: p = 0.317).

**Figure 1:**
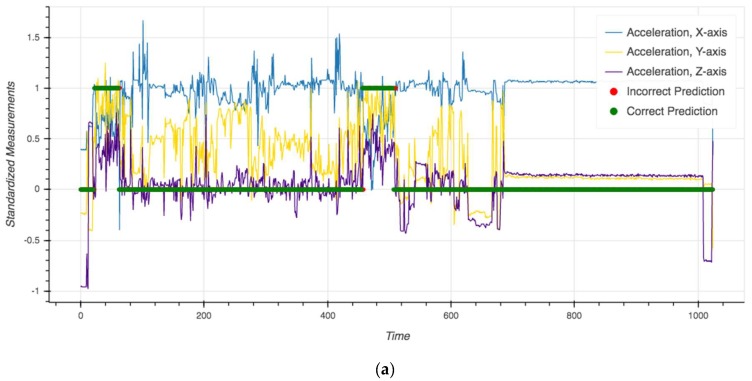

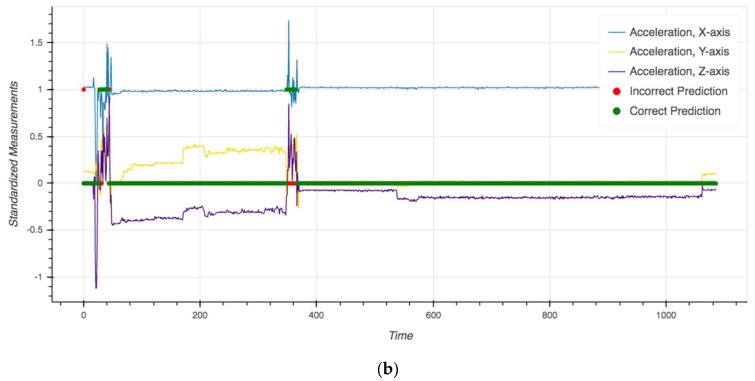
Model predictions and “ground truth” activities over time for the best performing LOSO fold (**a**) and worst performing LOSO fold (**b**). Green areas indicate a correct prediction and red areas indicate a misclassification. Each time unit on the X-axis represents a sliding window of six 30-second readings, with 50% overlap, that was used in the analysis.

Though the evaluation metrics are useful for exposing differences in predictions when class imbalance exists, they may obscure important relationships in the data that are useful for the final use case. To better understand where the models fail, Figure 1 depicts the mode smoothed transformer predictions over time for the session in which it performed the best (F1: 0.95) and worst (F1: 0.52). Areas in green indicate where the model correctly predicted biking (biking = 1, non-biking = 0), whereas areas in red indicate a misclassification. For additional context, we also included the accelerometer readings (X-, Y-, Z-axes) overlaid on the same plot. 

Although the model correctly identifies periods of biking for the vast majority of observations in both the best (99.0%) and worst (97.8%) performing sessions, it tends to struggle during transitions between activities. This error is magnified by the relatively high class imbalance in the worse performing session (only 3.1% of the observations labeled as “biking”) and the overall small number of biking examples (n = 34). Furthermore, given the difficulties in accurately labeling sensor data [27], activity transitions are also the portion of the manual annotations most prone to mislabeling, which may contribute to model misclassification rates.

### 4.2. Feature Importance

**Figure 2:**
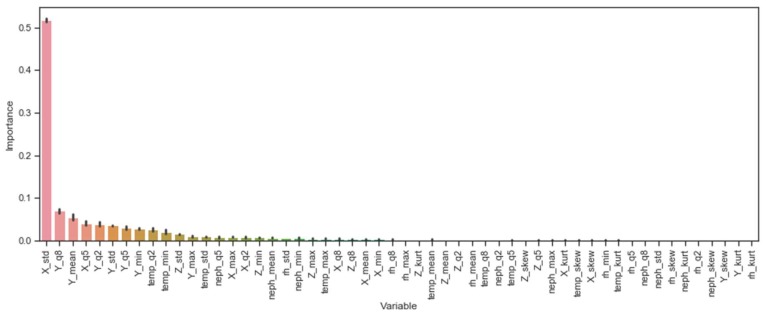
Normalized feature importance for the gradient boosted tree classifier. The naming convention for the abbreviations on the figure’s X-axis consists of (1) the measurement type recorded by the MicroPEM and (2) the summary statistics for observations contained within each sliding window. For example, “temp_kurt” is shorthand for the kurtosis of the temperature readings contained within the sliding window. The six MicroPEM measurements used in this study are: X-axis acceleration, Y-axis acceleration, Z-axis acceleration, temperature, relative humidity, and RH-corrected nephelometer readings. The nine summary statistics calculated are: mean, median, max, min, standard deviation, skew, kurtosis, 20% percentile, and 80% percentile.

To better understand which features are contributing to the model predictions, we calculated the normalized feature importance [20] for the gradient boosted tree classifier (Figure 2). To determine feature importance for tree-based supervised learning algorithms, the purity metric values normally used for determining the quality of splits at each node are combined to determine the total decrease in node impurity attributed to a given predictor. For tree ensembles, such as the gradient boosted trees classifier, these totals are averaged over all generated trees to generate final feature importance scores. To help with interpretability, the scores are normalized to sum to one. 

Though both the mode smoothed transformer and the stacked ensemble outperformed the gradient boosted tree, they both also use model predictions as inputs, complicating attribution to the original features. As the gradient boosted tree classifier is the best performing base model and an input to both the mode smoothed transformer and the stacked ensemble, we use it as a transparent proxy to help explain what features drive model performance.

The most important features tend to be associated with variables generated from the X-axis and Y-axis accelerometer readings, accounting for eight of the top 10 features and accounting for roughly 85.4% of the mean decrease in node impurity (X-axis: 58.7%; Y-axis: 26.7%). In terms of summary statistics, the standard deviation of values within the sliding windows tended to be most helpful in distinguishing between periods of biking and not-biking, with the percentile summary statistics also contributing greatly. For Y-axis acceleration values, in particular, higher values appear to be correlated with biking activity. For non-accelerometer derived features, the temperature and nephelometer readings were relatively more important (temperature 20% percentile, 9th; temperature minimum, 10th; temperature standard deviation, 13th; PM2.5 median, 14th). Across all sensor variables, relative humidity readings tended to be the least predictive when taking into account other factors (RH standard deviation, 19th).

## 5. Discussion

Our findings both confirm existing results in HAR modeling of biking activity, and suggest new approaches relevant for exposure assessment studies. First, in agreement with several studies [15,17,28], we found tree-based models to outperform other popular classification algorithms for HAR tasks, with KNN also performing well. Additionally, our model evaluation metrics for biking activity are comparable to other examples in the literature mentioned in Section 1.2, and we also found detecting crisp activity transitions to be challenging [27]. These findings should be generally encouraging for exposure scientists and environmental epidemiologists wanting to use wearable air quality monitors with built-in accelerometers for activity detection.

Our results also contribute uniquely to the HAR literature in several ways. First, our work can help researchers better understand what features best help distinguish biking from other activities that users would normally perform in their daily routines. In particular, changes in the X-axis acceleration and Y-axis acceleration by far contributed the most to uniquely predicting episodes of biking. While we hypothesized that non-accelerometer readings could help distinguish periods of biking (e.g., sharp change in temperature may indicate moving from indoors to outdoors, signaling different candidate activities), their contribution paled in comparison to the impact of the accelerometer readings. To our knowledge, this outcome has not been documented, particularly for studies monitoring subjects in naturalistic, non-lab settings in which changes in temperature, humidity, and air-borne particulate concentrations could plausibly help with classification. Future research is needed to see if this relationship continues to hold when assessing otherwise similar activities but in different environments (e.g., indoor biking vs. bike commuting). Another interesting finding from this study is that using a relatively low sampling rate does not appear to severely impact the classification rate for biking. This provides further evidence that these methods are appropriate for exposure assessment studies, where subjects are expected to wear devices over extended periods of time under less than ideal conditions (from a data collection perspective). Lastly, while temporally smoothing predictions has previously been proposed in the literature (e.g., Yang [17] uses a hidden Markov model to determine contiguous phases of activity from raw predictions), our mode smoothed transformer approach has shown to be a simple and effective alternative that substantively improves HAR results in the presence of temporally sporadic prediction trends. This method benefits from being easy to implement and explain to various stakeholders, a nice feature given the diverse backgrounds of exposure assessment study consumers.

Though our results are promising, they also have several limitations. Due to our study population of interest (urban cyclists), we only attempt to identify periods of biking activity, whereas other studies consider upwards of a dozen different activity types. While we believe our results are reasonable, researchers may observe different levels of accuracy than those reported if additional activity groups are required for recognition. In other studies, biking was most often misclassified as riding an elevator [15], jogging [16], or driving [17]. However, we take some comfort that, across all studies reviewed, machine learning approaches were able to distinguish biking from other activities at high rates, even in the presence of many other activity classes. While our study provides evidence for this approach in a naturalistic urban setting, it only captures subject routines occurring in fall months and may not extend as well to indoor physical activities that are more common in other seasons (e.g., running on a treadmill indoors during winter months). Our study is limited by the number of sessions and unique subjects. While not uncommon in HAR studies to have relatively large numbers of observations for a few subjects, we acknowledge that more participants and sessions should help capture greater inter-class variations in how activities are performed. Lastly, we did not explore the use of deep learning models for HAR, though they have shown much recent promise [29]. Though deep learning approaches were not pursued mainly due to our limited labeled training data, future research could explore the use of transfer learning [30] to determine if pre-trained neural networks can help detect patterns with modestly sized training sets, even with devices, sampling rates, and activity classes that differ greatly from what the pre-trained network was originally trained on.

## 6. Conclusions

Using machine learning, we demonstrate that personal pollutant exposure devices equipped with accelerometers can identify periods of biking with reasonable accuracy. This finding helps strengthen the case for using wearable monitors in exposure assessment studies, as it becomes possible to estimate potential inhaled dose if personal exposure data is combined with respiration rate. However, it should be noted that the most predictive features of our biking activity model were accelerometer readings, suggesting that personal pollutant exposure monitors without accelerometers may struggle to consistently identify periods of biking with atmospheric sensors alone. While this study focuses on biking activity, a reasonable next step would be accurately determining other activities of high excursion directly from wearable exposure monitor data.

## Figures and Tables

**Table 1:** Study summary statistics across all 21 sessions.

Statistics Per Session	Mean	Median	Min	Max
Records	992	989	916	1087
Percent Biking	7.4%	6.7%	3.1%	11.8%
X-axis acceleration (m^2^/s)	0.84	0.95	–0.95	2.26
Y-axis acceleration (m^2^/s)	0.06	0.02	–1.81	1.33
Z-axis acceleration (m^2^/s)	0.02	0.03	–1.37	1.82
Temperature (C)	24.8	24.4	9.0	33.5
Relative Humidity (%)	50.0	50.2	17.9	94.5
PM2.5 (µg/m^3^)	22.4	3.0	0.0	1640.0

**Table 2:** Model evaluation metrics for each model. The numbers reported are the mean and standard deviation across the 21 LOSO CV hold-out folds [mean (std)] for both weighted and unweighted metric variants. The best results per metric are presented in bold.

Model	F1		Precision		Recall	
	Raw	Weighted	Raw	Weighted	Raw	Weighted
Mode Smoothed	**0.832**	**0.979**	**0.884**	**0.980**	0.801	**0.980**
	**(0.117)**	**(0.011)**	**(0.063)**	**(0.011)**	(0.166)	**(0.010)**
Stacked Ensemble	0.767	0.970	0.791	0.971	0.767	0.970
	(0.115)	(0.012)	(0.115)	(0.011)	(0.115)	(0.012)
Gradient Boosted Trees	0.746	0.969	0.810	0.970	0.714	0.970
	(0.151)	(0.011)	(0.103)	(0.010)	(0.193)	(0.010)
K-Nearest Neighbors	0.744	0.965	0.780	0.967	0.732	0.965
	(0.115)	(0.017)	(0.137)	(0.015)	(0.138)	(0.018)
Logistic Regression	0.656	0.936	0.542	0.961	**0.909**	0.923
	(0.141)	(0.038)	(0.182)	(0.015)	**(0.074)**	(0.051)

## Appendix A

**Table A1:** Final hyperparameter specifications for the models used in this study.

Model	Specification
Mode Smoothed	Window length: 11 Window orientation: Centered on current observation
Stacked Ensemble	Classifier type: Gradient boosted trees classifier Features: Base model predicted probabilities Number of boosting stages: 40 Learning rate: 0.1
Gradient Boosted Trees	Number of boosting stages: 80 Learning rate: 0.1
K-Nearest Neighbors	K: 5 Weight function: Points weighted by inverse of their distance Distance metric: Euclidean
Logistic Regression	Regularization: L1-norm C (inverse of regularization strength): 0.0008 Class weights: balanced Optimization Solver: SAGA [31]
